# One-year marginal bone loss and survival rate of triangular-neck dental implants: a systematic review and meta-analysis

**DOI:** 10.1186/s40729-026-00702-5

**Published:** 2026-07-11

**Authors:** Ziqi Xie, Sara Reda Sammour, Ryuji Shigemitsu, Nobuhiro Yoda, Toru Ogawa

**Affiliations:** 1 Division of Advanced Prosthetic Dentistry, Tohoku University Graduate School of Dentistry, Sendai, Japan; 2 Division of Comprehensive Dentistry, Graduate School of Dentistry, Tohoku University Graduate School of Dentistry, 4-1 Seiryo-machi, Aoba-ku, Sendai, 980-8575 Japan; 3 Department of Prosthodontics, Faculty of Dentistry, Tanta University, Tanta, Egypt; 4 Liaison Center for Innovative Dentistry, Division for Advanced Education Development, Tohoku University Graduate School of Dentistry, Sendai, Japan

**Keywords:** Dental implants, Dental prosthesis design, Triangular-neck implant, Marginal bone loss, Implant survival

## Abstract

**Purpose:**

This systematic review aimed to evaluate the clinical performance of dental implants with a triangular neck, focusing on 1-year marginal bone loss (MBL) and implant survival rate.

**Methods:**

This review was conducted in accordance with the Preferred Reporting Items for Systematic Reviews and Meta-Analyses (PRISMA) guidelines. A comprehensive search of databases (PubMed, Scopus, and Web of Science) was completed on June 8, 2025. Randomized controlled trials (RCTs), retrospective and prospective studies, and case series that studied ≥ 10 implants, reporting on triangular-neck implants, were included. The Cochrane risk of bias 2 tool was used for RCTs, and the Joanna Briggs Institute case series checklist was used for nonrandomized studies. The results were summarized using a random-effects meta-analysis and sensitivity analysis was performed to assess robustness. The Grading of Recommendations Assessment, Development, and Evaluation (GRADE) method was used to determine the certainty of evidence.

**Results:**

The search provided 115 titles after removal of duplicates. Eight studies met the inclusion criteria, five of which provided sufficient data for quantitative synthesis. The pooled analysis demonstrated a 1-year mean MBL of 0.35 mm (95% confidence interval [CI]: 0.24–0.46; heterogeneity; I^2^ = 23.2%; *p* = 0.25) and an implant survival rate of 97.8% (95% CI: 95.4%–99.4%; I^2^ = 0.0%; *p* = 0.96).

**Conclusions:**

Available single-arm evidence indicates that triangular-neck implants were associated with high implant survival rates and limited marginal bone level changes during the first year of follow-up. However, the current evidence does not permit conclusions regarding comparative clinical benefits relative to other implant designs.

**Supplementary Information:**

The online version contains supplementary material available at https://doi.org/10.1186/s40729-026-00702-5.

## Background

Dental implant therapy has become a widely accepted treatment option for replacing missing teeth, with high long-term survival rates in various clinical situations [1]. Despite these favorable outcomes, preservation of the peri-implant marginal bone remains a critical determinant of long-term implant success because excessive marginal bone loss (MBL) can compromise both functional stability and esthetic outcomes [2].

Numerous influencing factors for peri-implant bone remodeling have been identified, including implant design, surgical technique, loading protocol, and patient-related variables; these affect the stability of the implant–bone interface, distribution of functional forces, and maintenance of surrounding soft and hard tissues. These factors determine the long-term success of dental implants [3].

Conventional circular-neck implants generally have a circular cross-section; however, implants with alternative neck geometries, such as triangular, conical, or platform-switched, have been developed to optimize stress distribution at the crestal bone and support peri-implant soft tissues [4].Triangular-neck implants reduce the contact area at the cortical crest and redistribute the occlusal forces, potentially minimizing MBL. Evidence from finite element analyses indicates that triangular, three-point support designs at the implant–abutment interface can reduce stress concentration [5]. These results highlight the biomechanical advantages of triangular configurations and may inform future design improvements in implant neck geometry.

Several clinical studies have investigated the performance of triangular-neck implants and reported outcomes, such as marginal bone loss, peri-implant soft tissue stability, and implant survival rates. However, these studies varied considerably in terms of methodologies and clinical contexts. Differences exist in terms of study design (prospective vs. retrospective), follow-up duration (ranging from 1 to 5 years), and the number of implants assessed. Moreover, triangular-neck implants have been evaluated in both single-tooth and multiunit restorations, in the anterior and posterior regions, and under immediate and conventional loading protocols. Variations in prosthetic protocols and radiographic measurement methods further contribute to the heterogeneity of reported outcomes.

Consequently, some investigations suggest that the triangular neck design may reduce crestal bone remodeling and enhance peri-implant tissue stability [6], whereas others report only minimal differences, compared with conventional circular neck designs [7]. To date, no systematic review has comprehensively evaluated the available clinical evidence on the performance of triangular-neck implants. Therefore, this systematic review and meta-analysis aimed to evaluate the clinical outcomes of triangular-neck implants in adults, with marginal bone loss (MBL) defined as the primary endpoint and overall implant survival rate as the secondary outcome.

## Methods

This review was performed in accordance with the guidelines of the Preferred Reporting Items of Systematic Reviews and Meta-Analyses and the Population, Intervention, Comparison, Outcomes, and Study (PICOS) criteria. The review protocol was registered in PROSPERO (# CRD420251090626).

### Specific question

In adults receiving triangular-neck implants, what are the clinical outcomes in terms of marginal bone loss and implant survival after at least 12 months of follow-up?

### Eligibility criteria

The PICOS criteria were used for the inclusion and exclusion of studies, as follows: Population (P): Adults (aged ≥ 18 years) receiving triangular-neck implants; intervention (I): Placement of triangular-neck implants; comparison (C): Not applicable; both single-arm and comparative studies were included; outcomes (O): Primary outcome: Marginal bone level change after at least 12 months of follow-up; Secondary outcome: Implant survival rate.

Inclusion criteria: adult patients (aged ≥ 18 years) treated using triangular-neck implants; studies reporting marginal bone loss at ≥ 12 months of follow-up; RCTs, prospective and retrospective cohort studies, and case series that studied ≥ 10 implants; and studies published as full-text articles in peer-reviewed journals.

Exclusion criteria: animal studies, in vitro experiments, finite element analysis, and case reports; studies with follow-up periods < 12 months; studies that did not report marginal bone loss; reviews, editorials, conference abstracts, and gray literature; and non-English language publications.

### Search strategy and data collection

A systematic literature search of the following databases was performed: PubMed, Scopus, and Web of Science. The latest search was conducted on June 8, 2025. The detailed search was performed using Medical Subject Heading terms and free text, which are listed in Table 1. The reference lists of the included studies and relevant reviews were screened to identify additional eligible articles.

**Table 1:** Search strategy

Data base	Search strategy
Web of science	TS=(“triangular” OR “three sided” OR “triangular cross-section” OR “triangular profile” OR “triangular shape” OR “three-cornered”) AND TS=(“implant neck” OR “implant collar” OR “implant shoulder” OR “cervical portion” OR “dental implant” OR “oral implant” OR “endosseous implant” OR “implant design” OR “implant macrogeometry”)
PubMed	(“triangular” [tiab] OR “three sided” [tiab] OR “triangular cross-section” [tiab] OR “triangular profile” [tiab] OR “triangular shape” [tiab] OR “three-cornered” [tiab]) AND (“implant neck” [tiab] OR “implant collar” [tiab] OR “implant shoulder” [tiab] OR “cervical portion” [tiab] OR “dental implant” [tiab] OR “Dental Implants” [Mesh] OR “oral implant” [tiab] OR “endosseous implant” [tiab] OR “implant design” [tiab] OR “implant macrogeometry” [tiab]
Scopus	TITLE-ABS-KEY ( “triangular” OR “three sided” OR “triangular cross-section” OR “triangular profile” OR “triangular shape” OR “three-cornered” ) AND TITLE-ABS-KEY ( “implant neck” OR “implant collar” OR “implant shoulder” OR “cervical portion” OR “dental implant” OR “oral implant” OR “endosseous implant” OR “implant design” OR “implant macrogeometry” )

All retrieved records were imported into Mendeley and duplicates were removed. The titles and abstracts were independently screened by two reviewers (Z. X. and S. R. S.) and full-text assessments were conducted for potentially eligible studies. Discrepancies were resolved through discussion or consultation with a third reviewer (T. O.).

Studies were included in the review based on the inclusion and exclusion criteria. Data of interest were independently extracted by two reviewers (Z. X. and S. R. S.) according to general study characteristics (authors, year of publication, and type of study), patient characteristics (sample size, age, sex, inclusion and exclusion criteria), implant characteristics (brand, diameter, length, placement protocol, and insertion site), and outcome measures. The outcome measures included marginal bone level, pink esthetic score and implant survival rate. Any disagreements between the two reviewers during the data extraction process were resolved through consensus or via consultation with a third reviewer (T. O.).

### Assessment of risk of bias and certainty of evidence of the included studies

The risk of bias of the included studies was assessed according to their study design. RCTs were evaluated using the Cochrane risk of bias 2 tool, which examines five domains: bias arising from the randomization process (D1), bias due to deviations from intended intervention (D2), bias due to missing outcome data (D3), bias in outcome measurement (D4), and bias in the selection of reported results (D5). Based on the sum of the scores assigned for the individual questions, the tool classifies studies as having a low risk of bias, some concerns, or a high risk of bias [8]. An overall risk-of-bias judgment was then assigned according to the risk of bias 2 algorithm. The web application “robvis” was used to graphically display the risk of bias assessment results for each study. Observational studies, including single-arm prospective or retrospective studies and case series, were assessed using the Joanna Briggs Institute (JBI) case series checklist, which comprised 10 items evaluating the clarity of inclusion criteria, reliability of condition measurement, validity of methods used to identify the condition, consecutive and complete inclusion of participants, reporting of demographic and clinical information, clarity of outcome reporting, adequacy of follow-up, and appropriateness of statistical analysis, each rated as “yes,” “no,” “unclear,” or “not applicable.” A score of 1 was assigned for each “yes” response and 0 for all other responses. The total score ranged from 0 to 10, with studies scoring 8–10 considered low risk of bias, 5–7 considered moderate, and 0–4 considered high risk. Two reviewers (Z. X, S. R. S) independently performed the assessment, and disagreements were resolved through discussion or consultation with a third reviewer (T. O). The results are summarized narratively and presented in tables and figures to provide an overview of the methodological quality of the included studies.

GRADEpro GDT (https://gradepro.org/) and the guidance provided in Chap. 14 of the Cochrane Handbook for Systematic Reviews of Interventions (Handbook 2021) [9, 10] were used to assess the certainty of evidence for MBL and implant survival. The certainty of evidence was evaluated based on risk of bias, inconsistency across studies, imprecision of findings, and indirectness. In this review, the resulting certainty ratings reflect confidence in the observed outcomes rather than comparative treatment effects.

### Data synthesis

Data synthesis was performed to summarize the clinical outcomes of triangular-neck implants, focusing on marginal bone level change as the primary outcome and implant survival as the secondary outcome. Statistical analyses were performed using Jeffrey’s Amazing Statistics Program (JASP) (JASP Team, Amsterdam, Netherlands). Effect sizes were calculated as mean differences with corresponding standard errors for each study. The choice between fixed-effect and random-effects models was predetermined based on clinical plausibility rather than statistical testing. Given the inherent clinical variations among the included studies, such as differing loading protocols, surgical expertise, and patient baseline characters, a single common true effect size could not be assumed across different settings. Therefore, a random-effects model was prospectively employed to appropriately encompass this methodological diversity and provide more conservative, generalizable pooled estimates.

Forest plots were generated to illustrate the effect size of each study, together with the pooled estimate and 95% confidence interval (CI). Between-study heterogeneity was assessed using Cochrane’s Q test, T^2^, and the I^2^ statistic. A Q test* p*-value < 0.1 was considered indicative of significant heterogeneity. Prediction intervals were calculated to reflect the expected range of effects in future studies.

Meta-regression analysis was performed to explore the potential sources of heterogeneity. Meta-regression models were fitted to examine whether the implant placement protocol and sample size influenced MBL outcomes. Additionally, a post-hoc leave-one-out sensitivity analysis was performed using RStudio (Version 4.4-0) to evaluate the numerical stability and robustness of the pooled MBL estimate by iteratively omitting each individual study.

## Results

### Study selection

**Fig. 1:**
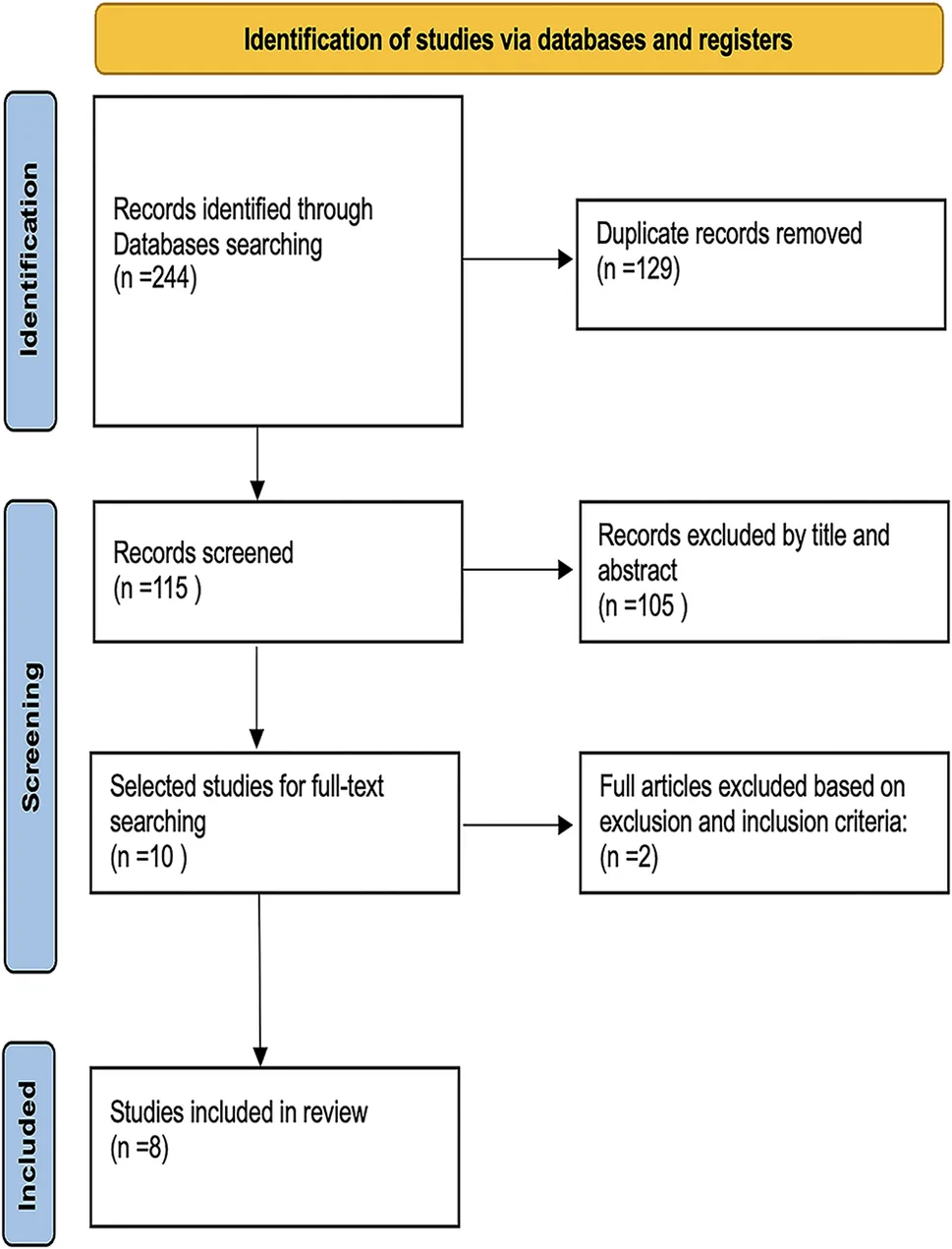
Preferred reporting items for systematic reviews and meta-analyses (PRISMA) flowchart

A total of 244 records were identified through the database search. After removing 129 duplicates, 115 records were screened based on titles and abstracts, of which 104 were excluded. The remaining 10 articles were assessed for full-text eligibility. Following the application of the predefined inclusion and exclusion criteria, 2 full-text articles were excluded, resulting in 8 studies included in the systematic review. The study selection process is illustrated in Fig. 1.

## Study characteristics

The main characteristics and clinical outcomes of the included studies are summarized in Tables 2 and 3. Comprehensive study information is available in Table S1. Eight studies published between 2018 and 2023 were included: four RCTs [6, 7, 11, 12], two prospective studies [13, 14], one retrospective study [15], and one case series [16]. In all the studies, triangular-neck implants (V3, MIS) were evaluated alone or compared with conventional circular-neck implants (C1, MIS).

**Table 2:** Characteristics of the included studies

Study	Study type	Study group	Sample size	Implant Brand	implant placement protocol	implant insertion site
Linkevicius [13]	Pros	According to vertical mucosal thickness, patients were assigned into three groups: Group 1 (thin, 2 mm or less): Group 2 (medium, 2.5 mm) Group 3 (thick,3 mm and more)	Group 1 (thin):19 Group 2 (medium):18 Group 3 (thick):18	V3	Healed site + without graft	Posterior maxilla and mandible
D’Avenia [15]	Retro	Triangular-neck implant group	20	V3	Immediate + with graft	Anterior maxilla
Li Manni [11] (baseline) Hurtgen [7] (follow-up study)	RCT	TN group RN group	TN group:17 RN group:17	Tri group: V3 Cir group: C1	Healed site + without graft	Posterior maxilla
Tokuc and Kan [12] (baseline) Tokuc and Kan [6] (follow-up study)	RCT	TN group RN group	TN group:20 RN group:20	TN group: V3 RN group: C1	Healed site + without graft	Anterior mandible
Collins [16]	CS	Triangular-neck implant group	25	V3	Healed site + without graft	Anterior & posterior, maxilla & mandible
Giralt-Hernando [14]	Pros	Triangular-neck implant group	120	V3	Healed site/ Immediate; with/ without graft	Anterior & posterior, maxilla & mandible

Pros, prospective study; Retro, retrospective study; RCT, randomized controlled trial; CS, case series; TN, triangular-neck implant; RN, regular (conventional) circular-neck implants; V3, V3 implant system; C1, C1 implant system

**Table 3:** Summary of the Clinical outcomes of the included studies

Study	follow-up time	Hard tissue measurement		Survival Rate (%)
		MBL measurement method	MBL (mm)	
Linkevicius [13]	1 year	Intraoral radiographs	Group 1 (thin): 1.25 ± 0.8 Group 2 (medium): 0.98 ± 0.06 Group 3 (thick): 0.43 ± 0.37	100
D’Avenia [15]	1 year	CBCT	Mesial: 0.09 ± 1.14 distal:0.13 ± 1.00 Average: 0.11 ± 1.00	100
Li Manni [11] (baseline) Hurtgen [7] (follow-up study)	1 year; 3 years; 5 years	CBCT	At 1 year: TN group: 0.22 ± 0.30 RN group: 0.42 ± 0.67	at 1year: 100 at 3 years: 100 at 5 years: 100
Tokuc and Kan [12] (baseline) Tokuc and Kan [6] (follow-up study)	1 year; 5 years	CBCT	At 1 year: TN group: 0.5 ± 0.3 RN group: 0.91± 0.59	at 1year: 100 at 5 years: 100
Collins [16]	15.6 m	Periapical radiogragh	Mesial: 0.14 ± 0.66 distal: 0.21 ± 0.74	100
Giralt-Hernando [14]	1 year	Periapical radiogragh	Mesial: 0.51 ± 0.44 distal: 0.52 ± 0.50 average: 0.51 ± 0.44	97.5

TN, triangular-neck implant; RN, regular (conventional) circular-neck implants; CBCT, cone-beam computed tomography

The total sample comprised 339 implants placed in partially or fully edentulous patients, with the mean patient age ranging from 45–61 years. Both male and female patients were included, with most studies enrolling systemically healthy adults (American Society of Anesthesiologists physical status I–II) and excluding individuals with uncontrolled systemic diseases, heavy smokers, and those taking medications affecting bone metabolism.

The implant placement protocols varied. Most studies investigated implants in healed ridges without grafting, whereas some included immediate placement with or without simultaneous augmentation. The placement sites were located across the anterior and posterior regions of both jaws. The implant diameters varied between 3.0 and 5.0 mm and lengths varied between 8 and 16 mm. The follow-up period ranged from 12 months to 5 years.

MBL were assessed radiographically using either periapical radiography or cone-beam computed tomography (CBCT). After 1 year, the mean bone loss varied from 0.2–1.2 mm, with triangular-neck implants generally showing reduced bone remodeling, particularly in sites with thick mucosa or in the anterior region. The pink esthetic score (PES) was evaluated in three studies, revealing stable or improved outcomes over time. The implant survival rates were consistently high, ranging from 97.5% to 100%. A slightly lower survival rate (97.5%) was reported in a large prospective study, wherein implant loss was associated with smoking.

Blinding was rarely reported, although RCTs described patient-level allocation concealment. Across all studies, triangular-neck implants demonstrated peri-implant bone preservation comparable to or more favorable than that of circular-neck implants, without significant differences in soft tissue health or patient-reported outcomes.

### Risk of bias assessment

**Fig. 2:**
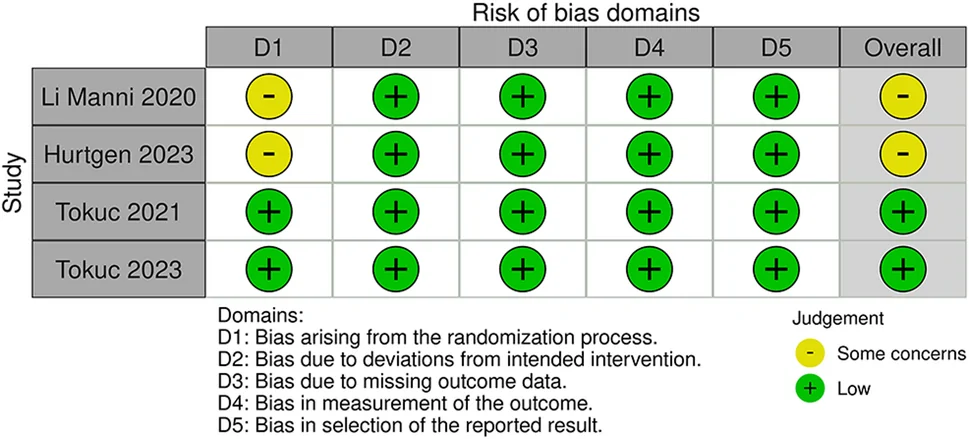
Risk of bias assessment for randomized controlled trials

The risk of bias assessment results for RCTs are summarized in Fig. 2, whereas those for nonrandomized studies are presented in Table 4. None of the RCTs [6, 7, 11, 12] were found to have a high risk of bias. Most studies were rated as having a low risk for deviations from intended intervention, missing outcome data, outcome measurement, and selection of reported results. Hurtgen et al. [7] and Li Manni et al. [11] were rated as having some concerns regarding the randomization process due to limited reporting on allocation concealment and were therefore judged to have an overall risk of some concerns. The other two trials by Tokuc and Kan [6, 12] were considered to have an overall low risk of bias.

**Table 4:** Risk of bias assessment for non-randomized studies

Study	Linkevicius [13]	D’Avenia [15]	Collins [16]	Giralt-Hernando [14]
Were there clear criteria for inclusion in the case series?	Y	Y	Y	Y
Was the condition measured in a standard, reliable way for all participants included in the case series?	Y	Y	Y	Y
Were valid methods used for identification of the condition for all participants included in the case series?	Y	Y	Y	Y
Did the case series have consecutive inclusion of participants?	U	U	U	U
Did the case series have complete inclusion of participants?	Y	Y	Y	Y
Was there clear reporting of the demographics of the participants in the study?	Y	Y	Y	Y
Was there clear reporting of clinical information of the participants?	Y	Y	Y	Y
Were the outcomes or follow up results of cases clearly reported?	Y	Y	Y	Y
Was there clear reporting of the presenting site(s)/clinic(s) demographic information?	Y	Y	Y	Y
Was statistical analysis appropriate?	Y	Y	Y	Y
Overall score	9	9	9	9
overall bias	low	low	low	low

Y, Yes; N, No; U, Unclear; N/A, Not applicable

For non-randomized clinical studies [13–16], the risk of bias was assessed using the JBI case series checklist. All studies clearly reported the inclusion criteria, measurement and identification of the condition, participant demographics, clinical information, outcomes, presenting site(s), and clinic(s) demographics. Consecutive inclusion of participants was unclear in all studies. Complete inclusion and statistical analyses were adequately reported. Overall, the methodological quality of the case series was high, with lack of clarity regarding consecutive inclusion being the main limitation.

### Synthesis of results

Among the eight eligible studies, five provided sufficient data for quantitative synthesis, whereas three were excluded from the meta-analysis because of incomplete reporting of MBL or survival outcomes at 1 year.

**Fig. 3:**
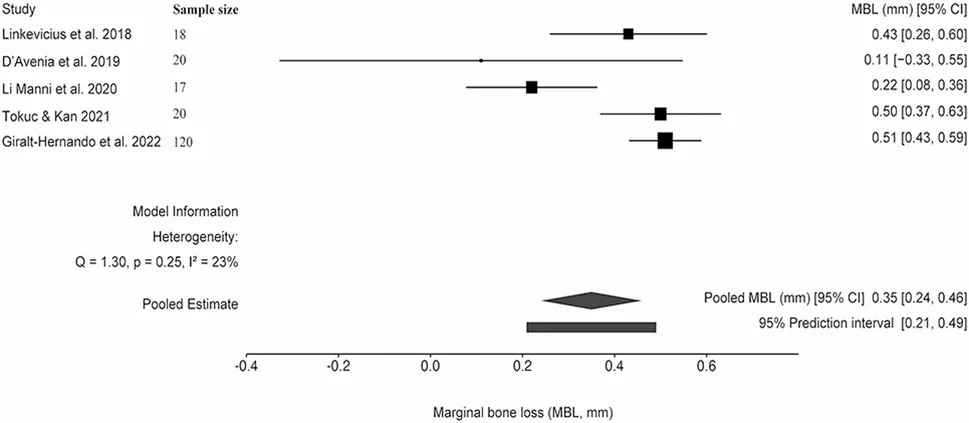
Forest plot of 1-year marginal bone loss (MBL)

For MBL (5 studies, 195 implants), the pooled effect size was 0.35 mm (95% CI, 0.24–0.46; *p* < 0.001), with low heterogeneity (Q = 1.30; *p* = 0.25; I^2^ = 23.2%; T^2^ = 0.002) and a 95% prediction interval of 0.21–0.49 mm (Fig. 3). Meta-regression demonstrated that the included moderators collectively explained a significant proportion of the between-study variability (omnibus moderator test: Qm = 9.50, df = 3, *p* = 0.023). Specifically, placement protocol and sample size were identified as significant moderators (*p* = 0.045 and *p* = 0.026, respectively). After accounting for these moderators, no significant residual heterogeneity remained (Qe = 1.30, df = 1, *p* = 0.254). Additionally, leave-one-out sensitivity analysis confirmed the overall numerical stability of the pooled estimate (Figure S1).

**Fig. 4:**
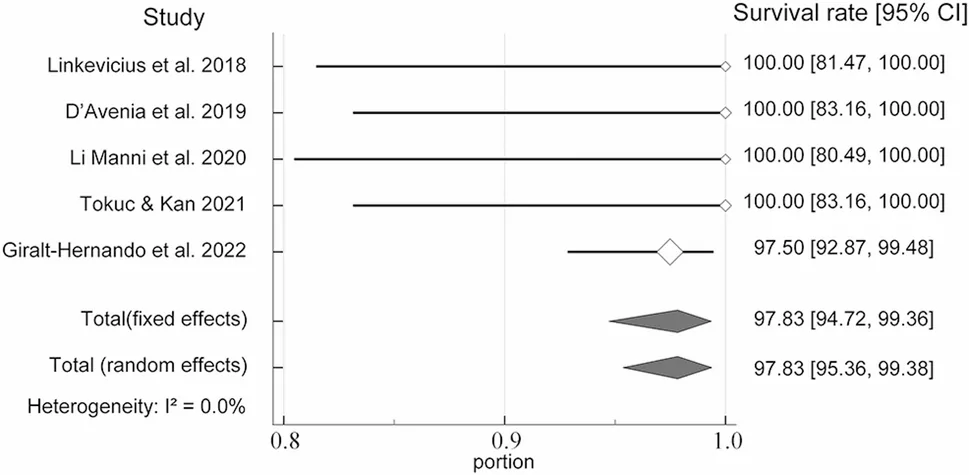
Forest plot of 1-year implant survival rate

The second outcome, the implant survival rate, was analyzed using the data from 5 studies on 195 implants. The pooled survival rate was 97.8% (95% CI, 95.4–99.4%) when using fixed- and random-effects models (Fig. 4). The heterogeneity was negligible (Q = 0.63, df = 4, *p* = 0.96; I^2^ = 0.0%), indicating consistent results across studies.

Publication bias assessment showed a significant Egger’s test result (intercept = 0.842, 95% CI, 0.597–1.087; *p* = 0.001) but a nonsignificant Begg’s test result (Kendall’s Tau = − 0.111; *p* = 0.786).

### Certainty of evidence

The certainty of evidence for MBL and implant survival assessed using the GRADE approach is summarized in Table 5. For MBL, the certainty of evidence from two RCTs with 1-year follow-up and radiographic assessment was rated as low. The certainty was downgraded because of serious inconsistency and imprecision. The certainty of evidence from three non-randomized studies was rated very low because of serious inconsistency and imprecision.

**Table 5:** GRADE assessment of evidence certainty for MBL and implant survival

Certainty assessment							Certainty
№ of studies	Study design	Risk of bias	Inconsistency	Indirectness	Imprecision	Other considerations	
Marginal bone loss (MBL) RCT only							
(Follow-up: 1 years; assessed with: Radiographic measurement (mm)							
2	Randomised trials	Not serious	Serious	Not serious	Serious	None	Low
Marginal bone loss (MBL) non-RCT only							
(Follow-up: 1 years; assessed with: Radiographic measurement (mm)							
3	Non-randomised studies	Not serious	Serious	Not serious	Serious	None	Very low
Survival rate RCT only (follow-up: 1 years)							
2	Randomised trials	Not serious	Not serious	Not serious	Serious	None	Moderate
Survival rate non-RCT only (follow-up: 1 years)							
3	Non-randomised studies	Not serious	Not serious	Not serious	Serious	None	Very low

For implant survival, the certainty of evidence from two RCTs with 1-year follow-up was rated as moderate and was downgraded because of imprecision. The certainty of evidence from three non-randomized studies was rated as very low because of imprecision.

## Discussion

### Principal findings

This systematic review evaluated the clinical outcomes of triangular-neck implants. Eight studies with follow-up periods ranging from 12 months to 5 years were included. The pooled analysis demonstrated a mean MBL of 0.35 mm after 1 year, which is lower than the clinically acceptable threshold of 1.5 mm proposed by Papaspyridakos et al. [1]. Implant survival was also high, with a pooled estimate of 97.8%.

### Agreements and disagreements with previous literature

When contextualized within the wider literature, the prior reviews on standard circular-neck implants documented 1-year MBL values ranging from 0.46 to 0.85 mm [17–19]. While direct cross-trial statistical comparisons are precluded by the single-arm design, the 1-year MBL of 0.35 mm reported herein sits within a similar clinically satisfactory spectrum, remaining well below the classic Albrektsson success criterion of less than 1.5 mm marginal bone loss during the first year of function [20]. Similarly, the aggregated 1-year implant survival rate of 97.8% aligns with established benchmarks for conventional circular-neck implants, such as the MIS C1 system (96.15%) [21] and wider systematic cohorts (95.8%–100%) [22]. These outcomes serve as descriptive parameters for the triangular-neck cohorts from heterogeneous clinical observations, rather than an index of relative efficacy or geometric superiority. The available data provide an overview of the short-term outcomes observed for triangular-neck implants.

However, the observed clinical performance of triangular-neck implants varies across the documented studies. For example, Linkevicius et al. [13] observed minimal differences in peri-implant bone levels, regardless of the implant neck design. Similarly, Hurtgen et al. [7] found no significant differences in MBL or peri-implant soft tissue stability between implants with triangular- and circular-neck designs at 5 years of follow-up. These findings suggest that the clinical performance of triangular-neck implants may be context-dependent and influenced by factors such as mucosal thickness, implant placement protocol, and follow-up duration.

The esthetics of peri-implant soft tissue is a critical determinant of implant success. D’Avenia et al. [15] reported that immediate placement with grafting of triangular-neck implants significantly increased the PES, suggesting promising esthetic outcomes. The triangular neck may facilitate bone regeneration by creating space for the graft material and blood perfusion, although its specific contribution remains uncertain. Li Manni et al. [11] also reported high PES; however, the exclusion of ridge preservation and immediate placement cases made it difficult to isolate the effect of the triangular-neck implants.

### Interpretation of results

The observed peri-implant tissue response of triangular-neck implants may be explained by their biomechanical and biological mechanisms. The triangular neck design reduces the contact area with the cortical crest, thereby redistributing the occlusal forces and reducing the stress concentration [5]. This redistribution could contribute to reduced marginal bone remodeling. In immediate placement protocols, the triangular neck design may also facilitate buccal bone preservation and create additional space for graft material and vascularization, thus supporting esthetic soft tissue outcomes [23]. From a mechanical standpoint, although the insertion torque patterns differ from those of circular implants, long-term stability is not compromised, as demonstrated by comparable resonance frequency analysis values after initial healing [4].

However, the lack of significant differences reported in some studies [7, 13] warrants further investigation. There are several possible explanations for these findings. First, short-to-mid-term follow-up intervals may lack the temporal scope required to reveal clinically relevant differences in marginal bone levels. Second, patient-related factors, such as mucosal thickness, occlusal load, and oral hygiene, could attenuate the influence of implant neck design on bone remodeling. Third, the statistical and clinical heterogeneity observed across the literature is well accounted for by our findings. Notably, the meta-regression framework identified the placement protocol and sample size as significant sources of this between-study variability in the MBL analysis. The impact of the placement protocol is biologically plausible, given that immediate and delayed implant placements differ substantially in healing dynamics and peri-implant bone remodeling. Sample size was also a significant moderator, suggesting that smaller studies may be more susceptible to random variation and less precise effect estimates. Admittedly, the limited study volume within these meta-regression analyses inherently introduces a degree of statistical uncertainty. Therefore, these findings are best characterized as exploratory and warrant a conservative interpretation. Additionally, a leave-one-out sensitivity analysis indicated that omitting Li Manni et al. [11] led to an upward shift in the pooled MBL to 0.49 mm. This shift occurred because, among the small-sample studies, Li Manni et al. [11] carried a substantially higher statistical weight due to its remarkably small standard error, while simultaneously reporting a lower baseline MBL (0.22 mm). The main limitation of CBCT is the appearance of metal artifacts, or “noise,” which reduces visualization quality. However, CBCT and intraoral radiography have shown comparable performances, with measurement accuracy influenced by image quality and defect size [24]. Comparable findings have also been reported for MBL assessment, where CBCT and intraoral radiography demonstrated similar accuracies [25]. These limitations suggest that the long-term clinical outcomes of triangular-neck implants remain to be further elucidated and verified over extended observation periods.

### Methodological quality and strength of evidence

The risk of bias assessment demonstrated favorable profiles, with all non-randomized studies exhibiting a low risk of bias, and the randomized controlled trials showing two categorized as “low risk” and two as having “some concerns”. This “some concerns” rating arose primarily from the inability to blind clinicians and participants, which represents a practical limitation in implant-related clinical trials. However, when evaluated using the GRADE system, the certainty of evidence ranged from moderate to very low across endpoints, clustering predominantly within the low certainty spectrum. This lower certainty is inherently tied to the current baseline landscape of global literature regarding triangular-neck implants. Specifically, because the pooled estimates were derived from a single-arm meta-analysis with a small cumulative sample size, the evidence body was inevitably downgraded due to imprecision and potential publication bias. Consequently, while this synthesized evidence base represents the current best available data derived from the published literature in this clinical field, the lack of direct comparative arms and the small cumulative sample size mean these findings should be interpreted as descriptive clinical tendencies rather than definitive comparative therapeutic advantages over conventional circular-neck implants.

### Strengths and limitations

A strength of this review is its exclusive focus on clinical studies reporting outcomes for triangular-neck implants, which allowed a direct assessment of their performance. The inclusion of RCTs and prospective studies strengthened the validity of the findings. Heterogeneity across the studies was generally low, and subgroup analyses indicated that the placement protocol and sample size influenced the results.

However, this study has some limitations must be acknowledged. First, as the pooled estimates were derived from a single-arm meta-analysis, they should be interpreted as descriptive summaries of the clinical performance of triangular-neck implants and do not support inferences regarding their comparative performance relative to conventional circular-neck implants. Second, all the included studies investigated implants from a single manufacturer (MIS V3), restricting the generalizability of the findings. Third, the follow-up period was limited to short- and medium-term periods, precluding conclusions regarding long-term performance. Fourth, despite the statistically significant Egger’s test (*p* = 0.001), the limited study volume restricts the reliability of this assessment, leaving the potential confounding of small-study effects unruled out. Finally, reports on esthetic outcomes and patient-reported measures were inconsistent, and blinding procedures were rarely described.

### Future perspectives

Further high-quality RCTs with longer follow-up periods are required to confirm the durability of triangular-neck implants. Future studies should also evaluate implants from different manufacturers to test whether the observed outcomes are design-specific or system-dependent. Standardized reporting of esthetic outcomes and patient-centered measures is important to fully understand the clinical relevance of this design. This review shows that triangular-neck implants maintain stable marginal bone levels and acceptable survival rates at the one year follow-up. However, because the available evidence is limited and the overall quality of data is low, these findings cannot serve as a definitive clinical guideline. For clinical decision-making, these preliminary data suggest that triangular-neck implants may be considered a treatment option in clinical situations similar to those evaluated in the available studies. Therefore, the available evidence primarily supports the use of triangular-neck implants in single-tooth restorations under immediate or delayed placement protocols, whereas evidence for more complex clinical scenarios remains limited. Future comparative studies should directly evaluate whether the triangular-neck implants provide measurable advantages over conventional circular-neck implants under comparable clinical conditions. Moreover, advanced imaging and biomechanical analyses could help clarify how the triangular design of the neck of the implants influence stress distribution, peri-implant bone remodeling, and soft tissue stability in different clinical contexts.

## Conclusions

Available single-arm evidence indicates that triangular-neck implants were associated with high implant survival rates and limited marginal bone level changes during the first year of follow-up. Specifically, the pooled MBL was 0.35 mm (95% CI: 0.24–0.46) and the survival rate was 97.8% (95% CI: 95.4–99.4%). However, the current evidence does not permit conclusions regarding comparative clinical benefits relative to other implant designs. Future multicenter, comparative RCTs with extended follow-up periods are needed to further evaluate the long-term clinical efficacy and durability of triangular-neck implants.

## Supplementary Information

Below is the link to the electronic supplementary material.

Supplementary Material 1: Fig. S1 Leave-one-out sensitivity analysis for 1-year marginal bone loss (MBL)

Supplementary Material 2: Table S1 Supplementary characteristics of the included studies. RCT, Randomized Controlled Trial; non-RCT, Non-randomized clinical study HSD, honestly significant difference; p, p-value; BC, bone-to-implant contact height; PES, Pink Esthetic Score; MBL, marginal bone loss; PROMs, patient-reported outcome measures; CI, confidence interval; CBL, crestal bone loss; TN, triangular-neck implant; RN, round-neck implant; BBT, buccal bone thickness; PPD, probing pocket depth; PI, plaque index; GI, gingival index; SD, standard deviation; OR, odds ratio; BOP, bleeding on probing

## Data Availability

All data supporting the findings of this study are available within the paper and its Supplementary Information.

## Abbreviations

CBCT: Cone-beam computed tomography
CI: Confidence interval
GRADE: Grading of recommendations assessment, development, and evaluation
JASP: Jeffrey’s amazing statistics program
JBI: Joanna Briggs Institute
MBL: Marginal bone loss
PES: Pink esthetic score
PICOS: Population, intervention, comparison, outcomes, and study
PRISMA: Preferred reporting items for systematic reviews and meta-analyses
PROSPERO: International prospective register of systematic reviews
RCT: Randomized controlled trial
